# Community resilience to floods in the coastal zone for disaster risk reduction

**DOI:** 10.4102/jamba.v10i1.356

**Published:** 2018-04-23

**Authors:** Muzakar Isa, Fransiscus X. Sugiyanto, Indah Susilowati

**Affiliations:** 1 Faculty of Economics, Muhammadiyah University of Surakarta, Indonesia; 2 Faculty of Economics and Business, Diponegoro University, Indonesia

## Abstract

The northern coast of the Central Java province is considered to be the critical area of flood path. The area is vulnerable to floods because of incessant rain and/or sea-level rise, resulting in suffering to people and the deterioration of the ecosystem. A number of measures have been implemented to manage the problem of floods, although the results are not noteworthy. It is obvious that infrastructure capacity for flood control, community awareness and other multiple factors significantly contribute to averting the problem of flooding in the area. This study aimed to determine the level of flood-zone vulnerability, the level of community resilience to floods and the influence of vulnerability aspects on community resilience. Interviews were conducted to outline the resilience model. A quantitative method was employed to analyse the data. The results of this study indicated that the exposure aspect is the greatest variable in describing flood vulnerability. At the same time, the greatest variables determining community resilience are damages, followed by losses and personal casualties. Among the flood vulnerability aspects are the exposure and adaptive capacities that determine the community resilience of the northern coast of Central Java.

## Introduction

Recent data have shown the vulnerability of the coastal zone to floods. Indonesia is ranked sixth in the world with regard to exposure to flood risk (Isa 2016). In 1815 and 2015, there were 5233 instances of flooding, which accounted for approximately 38.99% of all the natural disasters in Indonesia (Badan Nasional Penanggulangan Bencana [BNPB] 2016). Central Java province has a relatively high frequency of floods, particularly in the northern coast of Central Java. During 2011–2015, there were 368 instances of flooding in Central Java, which indicated an increase in frequency. In addition, floods had numerous negative impacts. In Central Java, during 2011–2015, flooding resulted in 58 people being killed, 191 422 people being evacuated, an area of 31.012 ha and 139 km of roads being destroyed and 1104 houses being badly damaged (BNPB 2016).

Floods definitely have various consequences, including economic losses, which could have been worse if management steps were not prepared before, during and after the floods. Generally, adaptation and mitigation are carried out before flooding to reduce the consequences. This can reduce the probability and magnitude of the stimulus in addition to reducing vulnerability and enhancing resilience. However, even in the absence of adaptation and mitigation, the community would have the ability to cushion or reduce the impacts of floods through community resilience (Isa 2015).

Economic resilience refers to flood mitigation and adaptation that enable individuals and communities to avoid some of the potential damages and losses (Isa 2013). This can take place at the household level. In contrast to the pre-event character of mitigation, economic resilience emphasises the ingenuity and resourcefulness applied during and after the event. Also, while mitigation often emphasises new technology (e.g. warning) or institutions (e.g. insurance markets), resilience has greater behavioural emphasis. It focuses on the fact that individuals and organisations do not simply react passively when facing a flood.

The concept of risk describes the assessment of the frequency of occurrence and magnitude of consequences associated with hazard activity (Hood & Jones 1996). One advantage of this approach is that risk does not automatically imply the occurrence of negative outcomes. Hood and Jones (1996) points out that risk management typically involves some mixture of mitigation and adaptation, thereby conferring upon risk management models the potential to encapsulate perspectives that cover growth and risk.

The less prescriptive risk concept provides a more flexible framework for the conceptualisation and analysis of disaster, affords opportunities to consider a range of outcomes and facilitates thinking about disaster risk mitigation strategies in terms of either enhancing resilience and/or reducing vulnerability. Given the importance of the risk management concept within contemporary emergency management, extending the use of the model in this way will render the training and development of disaster workers consistent with the prevailing strategic and operational paradigm in emergency and disaster management. As a starting point, it was appropriate to examine the components of this model and their implications for understanding and managing disaster risk.

Conceptualisations of risk generally include vulnerability as a determinant of differences in individual susceptibility to negative hazard effects. Blaikie et al. (1994) defined vulnerability as the combination of characteristics of a person or group in terms of their capacity to anticipate, cope with, resist and recover from hazard impacts that threaten their life, well-being and livelihood. Indeed, this definition contains elements consistent with the concept of resilience. Notwithstanding, capturing the wealth of resources that could be used to adjust to disaster experience, and developing comprehensive models of disaster risk, suggests that resilience should be included as a discrete category within the model. The next step to consider was how vulnerability and resilience can be modelled within a risk management framework to provide a systematic basis for assessing growth and loss outcomes.

Flooding, which usually occurs regularly and suddenly, is a condition that can threaten and disrupt people’s lives. According to Harjadi et al. (2007) and BNPB (2010), a flood is defined as a state where water floods on low land around a river, as a result of the river’s inability to accommodate and stream water.

Flood-prone areas pose the risk of human casualties, and damage to and loss of property. Muller, Reiter and Weiland (2011), Wisner et al. (2004), Smit and Wandel (2006), Turner et al. (2003) and Brenkert and Malone (2005) explained that people living in a non-flood-prone zone are able to solve a disruption caused by a flood, while those who are in a flood-prone zone with no condition that threatens will have no disaster risk. This demonstrated that the flood risk represents the function of vulnerability and hazard. Cutter (1996) and Cutter (2000) describe vulnerability as a condition that hinders the ability of people in a certain area to cope with flooding. Vulnerability is dynamic in accordance with the conditions, systems and environment of a community. Douben (2006) and Smit and Wandel (2006) suggested that flood area vulnerability consists of three aspects: exposure, sensitivity and adaptive capacity.

The low level of flood risk represents a high level of community resilience (Isa 2015). Djalante and Frank (2011) described resilience as the ability to survive and cope with floods and handle problems post-flood which will eventually minimise the risk. The collective behaviour of the community in addressing floods represents the community resilience. A high level of community resilience is a product of community empowerment against floods. Because the level of resilience is a conception of produced efforts, community resilience to flooding can be considered as a product.

The purposes of this study were: (1) to determine the level of flood-zone vulnerability, (2) to determine the level of community resilience to floods and (3) to determine the influence of vulnerability aspects (exposure, sensitivity and adaptive capacity) on community resilience. The study was carried out in the northern coast of Central Java province.

## Research methods

### Study area

**FIGURE 1:**
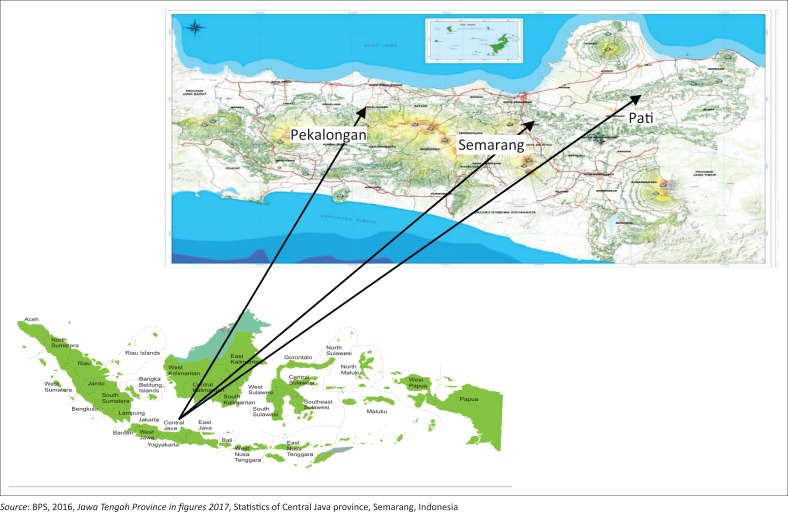
Location of the study area.

This study was conducted in Central Java province, Indonesia. Central Java is one of the 34 provinces of Indonesia. It has an area of 3 254 412 ha, constituting 1.70% of the total area of Indonesia. Central Java is bordered by the Indian Ocean and Yogyakarta to the south, West Java to the west, East Java to the east and the Java Sea to the north. Central Java is located between 5^°^40" and 8^°^30" south latitude and between 108^°^30" and 111^°^30" east longitude. More than 53% of the Central Java region is lowland. The lowlands lie on the north coast and the west coast. The northern coast is more vulnerable to flooding, which is caused by (1) high rainfall, (2) an overflow of rivers and (3) damage of dams and/or sluices. Flooding is more common in Pati Regency, Pekalongan and Semarang City. Several major rivers cross these areas, making them vulnerable to flooding. The overland function to residential area, agricultural expansion and industrial development on the lowlands contribute to the degradation of coastal areas in Pati, Pekalongan and Semarang City. This study was conducted in three sites that deputise the eastern, central and western areas of the northern coast of Central Java. These areas are Pati, Pekalongan and Semarang City. Figure 1 shows the location and geographic coordinates of the study area.

### Data processing and analyses

Vulnerability and community resilience are considered here based on the use of indicators. An indicator, or set of indicators, can be defined as an inherent characteristic that quantitatively estimates the condition of a system; they usually focus on minor, feasible, palpable and the telling piece of a system that can offer people a sense of the bigger representation. Therefore, it is very important to know the impacts on the people, cities and natural resources, via the use of these indicators.

The communities affected by floods in these three sites comprised the population of the study. Subsequently, a multistage sampling method was applied by using Slovin’s Formula. The number of respondents was 390. The respondents were interviewed directly using questionnaires. These questionnaires contained a list of questions that were used as a guide for the researchers to obtain respondents’ answers which formed the data.

Having chosen suitable indicators, these were normalised so as to bring the values of the indicators within the comparable range (Gbetibouo & Ringler 2009; Nelson et al. 2010; Vincent 2004). Normalisation is done by subtracting the mean from the observed value and dividing by the standard deviation for each indicator. Next, weights were assigned to these indicators.

The normalised variables were then multiplied by the assigned weights to construct the indices (separately for exposure, sensitivity and adaptive capacity), using the following formula (Chaliha 2012; Luni, Keshav & Dan Niraj 2012):

$$I_{j}=\sum_{i=1}^{k}b_{i}\left[\frac{a_{ji}-x_{i}}{s_{i}}\right]$$ [Eqn 1]

where ‘I’ is the respective index value, ‘b’ is the loadings from the first component from principal component analysis (PCA), taken as weights for respective indicators, ‘a’ is the indicator value, ‘x’ is the mean indicator value and ‘s’ is the standard deviation of the indicators.

A vulnerability index was established through the assessment of all aspects of resilience, covering exposure, sensitivity and adaptive capacity, as presented in Table 1. Vulnerability index formation region on flood assessment survey respondents carried through to all aspects of vulnerability, and the assessment results were then compiled. A greater value indicates that the level of vulnerability is getting smaller. The results of the data compilation examine every aspect of vulnerability, which are then normalised to obtain a score of 0–1 (Luni et al. 2012). To show the level of vulnerability region, the preparation is done by processing the vulnerability index score, which is 1 minus the result of the normalisation of the resultant data. The results showed that the higher the number (closer to 1), the higher the degree of vulnerability.

**TABLE 1:** Vulnerability variables and indicators.

Variable	Indicator	Definition
Exposure	Flood frequency	Number of years experiencing extremely high rainfall and severe floods taken as a proxy (number)
	Floodwater depth	Total depth of the floodwater (metres)
	Flood duration	Total amount of time the flood persisted in the village (days)
	Elderly	Percentage of household > 65 years old (%)
	Children	Percentage of household < 5 years old (%)
	Proximity to river	Total distance of the house from the river (metres)
Sensitivity	Health	Number of household members having health problems because of floods (number)
	Water availability during floods	Amount of freshwater to be purchased during floods (IDR)
	Income	Total income of the respondent (IDR)
	Migration	Number of families that migrated to town (number)
Adaptive capacity	Condition of river, embankments and sluices	Condition of river, embankments and sluices
	The availability of flood-prone maps	Availability of flood-prone maps (number)
	Education	Percentage of literate members in the household (%)
	Distance to the nearest health care centre	Distance travelled to the nearest public health centre (m)
	Evacuation sites	Distance travelled to reach the nearest evacuation site (m)
	Number of NGOs providing relief	Total number of NGOs providing relief to flood victims (number)
	Information access	Total access of flood information from television or mass media (number)
	Number of flood camps	Number of flood camps (number)
	Flood awareness	Percentage of household having assurance (%)
	Emergency services	Number of emergency services (number)
	Early warning of the flood	Early flood warning (number)
	Dissemination of flood prevention	Amount of dissemination on flood risk (number)
	Training of flood prevention	Amount of training on flood risk (number)

*Source*: Balica, Wright and Van der Meulen (2012), Chaliha (2012) and Weis et al. (2016)

Note: Please see the full reference list of the article, Isa, M., Sugiyanto, F.X. & Susilowati, I., 2018, ‘Community resilience to floods in the coastal zone for disaster risk reduction’, *Jàmbá: Journal of Disaster Risk Studies* 10(1), a356. https://doi.org/10.4102/jamba.v10i1.356, for more information.

IDR, Indonesian Rupiah, which is the currency of Indonesia; NGOs, nongovernmental organisations.

The next step is to do the weighting aspect vulnerability in consideration of the influence of each variable on the area above the flood vulnerability. If there is a higher influence of vulnerability index formation, it will result in a higher weight as well. Weighting was obtained through in-depth interviews with relevant stakeholders in the research sites. The results of the in-depth interviews showed the weight of the exposure to be 40%, the weight of adaptive capacity to be 35% and sensitivity to be 25%:

$$\begin{array}{l} \text{Vulnerability index}=\sum_{i=1}^{3}\left(W_{1}\times X_{1})+(W_{2}\times X_{2}\right)\\ \;+(W_{3}\times X_{3}) \end{array}$$ [Eqn 2]

Where Vulnerability index = Vulnerability index; W_1_ = Exposure Weight; X_1_ = Exposure Score; W_2_ = Sensitivity Weight; X_2_ = Sensitivity score; W_3_ = Adaptive Capacity Weight; X_3_ = Adaptive Capacity Score.

The vulnerability index is determined by multiplying the total score of all indicators and weights exposure variables, sensitivity and adaptive capacity. Vulnerability index results can be interpreted by three criteria: high vulnerability (index value ≥ 0.67), moderate vulnerability (index value between 0.34 and 0.66) and low vulnerability (index value ≤ 0.33). The vulnerability index is calculated using the formula in Equation 2 (Luni et al. 2012).

The community resilience to flood index was established through the assessment of all aspects of resilience, covering personal casualties, damage and losses, as presented in Table 2. The greater the value indicated a smaller resistance and vice versa. The results of data compilation of every aspect of this component were normalised to obtain a score of 0–1 (Luni et al. 2012). To demonstrate the level of community resilience, in the preparation of this community resilience index, this is done by processing the score; that is, 1 minus the result of the normalisation of the resultant data. The results showed that the higher the number (closer to 1), the higher the level of community resilience.

**TABLE 2:** Resilience variables and indicators.

Indicator	Definition
Damage (direct impact)	Flood damage. Damage includes (1) buildings and equipment (cars, motorcycles, furniture, electronics and other home supplies), (2) trade facilities (shops), (3) agricultural facilities (land and machinery – agricultural equipment), (4) farm facilities (cages and accessories), (5) fishing facilities, ponds or pools and equipment and (6) fishing equipment such as boats, engines, nets and more (IDR).
Loss (indirect impact)	Loss because of flooding. The losses include (1) trade (daily turnover multiplied by the number of days not operating), (2) agricultural (crop damage that caused a failed harvest and reduced yields), (3) loss because of the death of livestock, the type of cattle owned, (4) fisheries loss because of reduced incomes, (5) fisherman (loss of income because of the incapability to fish during floods) and (6) the type of work (loss because they cannot work because of flooding) (IDR).
Personal causality	The number of people who died, fell sick and/or were injured and/or migrated because of flooding (people)

*Source*: Bappenas, 2008. *Penilaian Kerusakan Dan Kerugian Bencana*, Bappenas, Jakarta

IDR, Indonesian Rupiah, which is the currency of Indonesia.

The next step was the measurement of the aspects of community resilience by considering the level of the resilience aspect. A higher weight meant a greater value of the endurance aspect. Weighting was obtained through in-depth interviews with relevant stakeholders in the research sites. The results of the interviews showed that the weight of the personal aspects of the victims was 40%, the weight aspect of the damage was 35% and the weight aspect was 25%:

$$\begin{array}{l} \text{Resilience index}=\sum_{i=1}^{3}\left(W_{1}\times X_{1})+(W_{2}\times X_{2}\right)\\ \;-(W_{3}\times X_{3}) \end{array}$$ [Eqn 3]

Where Resilience Index = Value of Resilience Index; W_1_ = Personal Causality Weight; X_1_ = Personal Causality Score; W_2_ = Damage Weight; X_2_ = Damage Score; W_3_ = Loss Weight; X_3_ = Loss Score.

A resilience index is determined by multiplying the score of all indicators and value aspects of personal casualties, damage and loss. As a result, a resistance index can be defined by three criteria: high resilience (index value ≥ 0.67), moderate resilience (index value between 0.34 and 0.66) and low resilience (index value ≤ 0.33). An index of resilience was determined by the formula below (Luni et al. 2012).

Multinomial logistic regression was used to analyse the effect of vulnerability index on flood risk level (resilience level). This method was assumed to be the appropriate tool because the dependent variable, the flood risk level, was multinomial or had more than two attributes: 3 for high risk level, 2 for medium and 1 for low.

Through multinomial logistic regression analysis, the empirical model of community resilience can be formulated as shown in the following equation:

$${\text{R}}_{\left(\text{it}\right)}=\alpha +\beta_{1}{\text{E}}_{\left(\text{it}\right)}+\beta_{2}{\text{S}}_{\left(\text{it}\right)}+\beta_{3}{\text{KA}}_{\left(\text{it}\right)}.$$ [Eqn 4]

Where α: Intercept; *β*_1_, *β*_2_…, *β*_3_: Coefficient; R_(it)_: Resilience variable; E_(it)_: Exposure variable; S_(it)_: Sensitivity variable; KA_(it)_: Adaptive capacity variable.

## Results and discussion

The level of flood-zone vulnerability in the northern coast of Central Java influenced the flood risk, including personal casualties (death, injury and evacuation), damages and losses. Flood risk showed that there were unsolved economic problems related to flood-zone vulnerability and community resilience to flood, which depicted an inefficiency in flood management.

The flood risk in a society portrays the ability of people to cope with flooding. The low flood ratio delineated that society was impervious to flooding. The flood risk consisted of personal casualties, damages and losses, referred to as ‘community resilience’. When floods occurred, the level of community resilience was determined by the level of its vulnerability.

### Flood-zone vulnerability

The vulnerability index of the northern coast of Central Java was 0.63. This index indicated the medium level of vulnerability, although the results were diverse in accordance with the city or district. Pekalongan district had the highest vulnerability level with an index of 0.67, which could be classified as a high vulnerability. Pati district and Semarang City were at a medium level, with indexes of 0.62 and 0.60, respectively.

Based on the results, Pekalongan district was the most vulnerable area compared with other sites, and it was classified as a place of high vulnerability. The result was similar to the Flood Risk Index released in 2014 by BNPB. The high vulnerability level in Pekalongan district was triggered by the high frequency of flooding, the floodwater level, the duration of the flood and the ineffective management of the local government. It was also influenced by human factors, as indicated by the fact that 79.4% of the local populace worked as farmers or fishermen. In addition, 81.5% of the respondents had only primary education (elementary and junior high school) and 76.3% of them earned ≤ 1 million Rupiah a month.

Table 3 indicates that the exposure variables and adaptive capacity were at a high vulnerability level. The two variables significantly contributed in determining the vulnerability level of the northern coast of Central Java, with index values of 0.81 and 0.73, respectively, that is, vulnerability index and resilience index. The sensitivity variable was classified in the medium vulnerability level, with an index of 0.36. The levels indicated that the government and society should pay more attention to the exposure variables which consisted of the flood frequency, flood duration, number of elderly and infants, and distance of settlements from the flood area.

**TABLE 3:** Index of flood area vulnerability in the northern coast of Central Java.

Location	Exposure		Sensitivity		Adaptive capacity		Vulnerability index
	Score	Weight	Score	Weight	Score	Weight	
1. Pekalongan	0.70	0.40	0.57	0.25	0.72	0.35	0.67
2. Semarang City	0.59	0.40	0.54	0.25	0.66	0.35	0.60
3. Pati	0.73	0.40	0.32	0.25	0.71	0.35	0.62
**Vulnerability index**	**0.81**		**0.36**		**0.73**		**0.63**

The causes of flood-zone vulnerability were divided into three aspects: flood aspects, local government service aspects and individual aspects. The flood and local government service were the external aspects of society. Therefore, local government and the community can mitigate the flood situation by creating rain infiltration, improving drainage, normalising the flow of the river, arranging buildings in accordance with applicable spatial plans and conducting institutional development. Institutional development could be achieved by strengthening the local disaster management agency, the development of standard operating procedures (SOPs) for floods and strengthening flood prevention management.

The flood and local government service were the external aspects of flood-zone vulnerability in the northern coast of Central Java. The aspects consisted of the flood frequency, water level and flood duration. In addition, the vulnerability level was also affected by the distance of the settlement from the river. It was also affected by a lack of local government services, such as (1) early flood warning, (2) dissemination of flood prevention, (3) training for flood prevention, (4) nongovernmental organisations involved in flood situations, (5) evacuation route, (6) the number of flood emergency services, (7) the distance of the evacuation site from settlements, (8) the number of aid camps for victims, (9) access to health services and (10) the condition of the river, embankments and sluices.

Based on the internal aspects of the community, the high vulnerability level to floods was caused by the low level of public awareness with regard to obtaining flood information as well as personal insurance. The low educational background and number of infants and the elderly were also contributory factors. Alternative solutions in the form of the dissemination of technology and knowledge was required to address the internal issue.

### Community resilience to flood

The index of the community resilience to floods in the northern coast of Central Java was 0.83. This indicated that people on the northern coast of Central Java had a relatively high resilience to floods. Based on the analysis, the community resilience indexes of Pekalongan district, Pati district and Semarang City, classified as high, were 0.89, 0.84 and 0.82, respectively.

Table 4 suggests that the damage aspect significantly represented the community resilience to flood, followed by the losses and personal casualty aspects. Damages in the northern coast of Central Java included (1) damage to buildings and equipment such as cars, motorcycles, furniture, electronics and other items, (2) trading facilities such as shops and kiosks, (3) agricultural facilities such as land and farm machinery, (4) livestock facilities such as farms and stables, (5) fishery facilities such as ponds or pools and (6) fishing equipment such as boats, engines and nets.

**TABLE 4:** Index of community resilience to floods in the northern coast of Central Java province.

Location	Personal casualties		Damages		Losses		Resilience index
	Score	Weight	Score	Weight	Score	Weight	
1. Pekalongan	0.72	0.33	0.97	0.33	0.98	0.34	0.89
2. Semarang City	0.72	0.33	0.92	0.33	0.83	0.34	0.82
3. Pati	0.65	0.33	0.96	0.33	0.92	0.34	0.84
**Resilience index**	**0.69**		**0.94**		**0.93**		**0.85**

The level of community resilience the northern coast of Central Java, based on the damages, was high. The value was in the range of between 0 and 1. The level of community resilience was linear to the value: the higher the value, the higher the community resilience to floods, and vice versa. The results of the analysis demonstrated that (1) in the trading sector, the amount of losses can be determined through the multiplication of trade turnover and the number of days off, (2) in the agricultural sector, the crop failure would automatically reduce crop yields, (3) in the livestock sector, it was obvious that floods increased the number of livestock deaths, (4) in the fisheries sector, the unfavourable conditions for fishermen would result in a reduced income, (5) fish was unavailable because of flood conditions, and (6) in other sectors, people could not carry on with their daily activities such as going to the workplace. However, the northern coast community had a high level of community resilience to floods.

The examination of the three locations showed different results, but in general, the number of evacuated victims as one of personal casualties was the lowest of index resilience. The number of injured and evacuated victims from Pekalongan district and Semarang City were almost similar, and Pati district had the highest number of evacuated victims.

The number of deaths because of floods in the northern coast of Central Java could be considered as low. However, flooding does not necessarily have an impact on deaths; injuries and evacuations are two common risks that result from flooding.

### Establishing community resilience to floods

There were three alternatives to community resilience to floods in the northern coast of Central Java, comprising high resilience, medium resilience and low resilience. The three alternatives were elicited from multinomial logistic regression by defining the variables of flood-zone vulnerability, including exposure, sensitivity and adaptive capacity as the independent variables.

Multinomial regression analysis models indicated that there was conformity with the data, which was indicated by the value of Pearson and deviance values. Pearson has a significant value of 0.374 and deviance had a significant value of 0.057. They explained that the significance of Chi-Square values was greater than 0.05; thus, it could be concluded that the model was fit to the empirical data.

The result of the Nagelkerke *R*^2^ is 0.081, which means that 8.1% of community resilience variables could be represented by exposure, sensitivity and adaptive capacity variables. Nevertheless, the remaining 91.9% was represented by the independent variables that were not included in the model.

The likelihood ratio test determined the significance of the model simultaneously. This was carried out by comparing the model (where the predictor variables were exposure, sensitivity and adaptive capacity) to community resilience. It obtained the significant value of 0.000. This means that the exposure, sensitivity and adaptive capacity variables simultaneously had a significant positive effect on the community resilience to floods in the northern coast of Central Java.

Tests on individuals were carried out to assess the parameter significance of predictor variables by using the likelihood ratio test. Based on this test, the significance values of exposure and adaptive capacity variables were less than 0.05; hence, it could be concluded that those two variables were positive and significant in affecting the community resilience to floods at α = 5%. The sensitivity variable was positive but insignificant in affecting the community resilience to floods at α = 5%.

Tests on individuals determined the parameter significance of predictor variables by using the Wald Test. The statistical test for the Wald Test is presented in Table 5.

**TABLE 5:** The Wald Test.

Response variable	Predictor variable	Wald	Significance	Beta	Expected (Beta)
Medium community resilience	Intercept	12.277	0.000	−6.135	-
	X1	3.251	0.071	0.217	1.242
	X2	0.926	0.336	0.083	1.087
	X3	7.151	0.007	0.105	1.110
High community resilience	Intercept	13.240	0.000	−5.832	-
	X1	10.745	0.001	0.354	1.425
	X2	1.655	0.198	0.099	1.104
	X3	0.777	0.378	0.032	1.032

The medium level of coastal community resilience to floods was affected by adaptive capacity variables (sig. 0.007) and exposure variables (sig. 0.071). The high level of community resilience was only affected by exposure variables (sig. 0.0001).

Exposure and adaptive capacity positively and significantly contribute to the community resilience to flood. From those two variables, the government can undertake several efforts to enhance community resilience, such as banning settlement construction in areas adjacent to the flood zone; stabilising the river, levees and floodgates; organising and conducting socialisation sessions of flood-prone areas on the map; improving the educational level of the community; building health services in the flood zone; establishing a precise evacuation route and/or path to the evacuation camp in the flood zone; encouraging the establishment of disaster relief nongovernment organisations; providing education to the community on information access; providing socialisation and education of hazard-insurance services; constructing a flood emergency camp during floods; providing early warning signs; and organising socialisation and training to address and relieve the hazard.

## Conclusion

Flood vulnerability is described by exposure, sensitivity and adaptive capacity. However, the exposure aspect is the greatest variable in describing the flood vulnerability of the northern coast of Central Java province. The exposure itself consists of the flood frequency, flood duration, number of elderly and infants, and distance of settlements from the flood area. At the same time, the greatest variables that determine the community resilience is damage, followed by losses and personal casualties. Among the flood vulnerability aspects, exposure and adaptive capacity determine the community resilience in the northern coast of Central Java.
